# Evidence of a Streamlined Extracellular Electron Transfer Pathway from Biofilm Structure, Metabolic Stratification, and Long-Range Electron Transfer Parameters

**DOI:** 10.1128/AEM.00706-21

**Published:** 2021-08-11

**Authors:** Fernanda Jiménez Otero, Grayson L. Chadwick, Matthew D. Yates, Rebecca L. Mickol, Scott H. Saunders, Sarah M. Glaven, Jeffrey A. Gralnick, Dianne K. Newman, Leonard M. Tender, Victoria J. Orphan, Daniel R. Bond

**Affiliations:** a BioTechnology Institute, University of Minnesota, Saint Paul, Minnesota, USA; b Department of Biochemistry, Molecular Biology, and Biophysics, University of Minnesota, Minneapolis, Minnesota, USA; c Division of Geological and Planetary Sciences, California Institute of Technology, Pasadena, California, USA; d Center for Bio-Molecular Science and Engineering, Naval Research Laboratory, Washington, DC, USA; e American Society for Engineering Education, Washington, DC, USA; f Division of Biology and Biological Engineering, California Institute of Technology, Pasadena, California, USA; g Department of Plant and Microbial Biology, University of Minnesota, Saint Paul, Minnesota, USA; University of Michigan—Ann Arbor

**Keywords:** *Geobacter sulfurreducens*, metabolic engineering, multiheme cytochromes, outer membrane electron conduit

## Abstract

A strain of Geobacter sulfurreducens, an organism capable of respiring solid extracellular substrates, lacking four of five outer membrane cytochrome complexes (*extABCD*^+^ strain) grows faster and produces greater current density than the wild type grown under identical conditions. To understand cellular and biofilm modifications in the *extABCD*^+^ strain responsible for this increased performance, biofilms grown using electrodes as terminal electron acceptors were sectioned and imaged using electron microscopy to determine changes in thickness and cell density, while parallel biofilms incubated in the presence of nitrogen and carbon isotopes were analyzed using NanoSIMS (nanoscale secondary ion mass spectrometry) to quantify and localize anabolic activity. Long-distance electron transfer parameters were measured for wild-type and *extABCD*^+^ biofilms spanning 5-μm gaps. Our results reveal that *extABCD*^+^ biofilms achieved higher current densities through the additive effects of denser cell packing close to the electrode (based on electron microscopy), combined with higher metabolic rates per cell compared to the wild type (based on increased rates of ^15^N incorporation). We also observed an increased rate of electron transfer through *extABCD*^+^ versus wild-type biofilms, suggesting that denser biofilms resulting from the deletion of unnecessary multiheme cytochromes streamline electron transfer to electrodes. The combination of imaging, physiological, and electrochemical data confirms that engineered electrogenic bacteria are capable of producing more current per cell and, in combination with higher biofilm density and electron diffusion rates, can produce a higher final current density than the wild type.

**IMPORTANCE** Current-producing biofilms in microbial electrochemical systems could potentially sustain technologies ranging from wastewater treatment to bioproduction of electricity if the maximum current produced could be increased and current production start-up times after inoculation could be reduced. Enhancing the current output of microbial electrochemical systems has been mostly approached by engineering physical components of reactors and electrodes. Here, we show that biofilms formed by a Geobacter sulfurreducens strain producing ∼1.4× higher current than the wild type results from a combination of denser cell packing and higher anabolic activity, enabled by an increased rate of electron diffusion through the biofilms. Our results confirm that it is possible to engineer electrode-specific G. sulfurreducens strains with both faster growth on electrodes and streamlined electron transfer pathways for enhanced current production.

## INTRODUCTION

The large diversity of microbial metabolic strategies has inspired many biotechnological applications, but only after gaining a mechanistic understanding of each pathway can these tools be manipulated and exploited. Microbial respiration of extracellular substrates is a metabolic strategy that can be harnessed to support technologies such as water desalination (1, 2), wastewater treatment (3), electrofermentation (4, 5), and bioproduction of electricity (6, 7). These biotechnological applications could be cost-effective and competitive with current alternatives if the power output could be increased ∼10× (3, 8). While recent research has revealed key proteins that microorganisms use to direct electrons from the cytoplasmic oxidation of organic acids to the reduction of substrates in the extracellular space, much less is known about how complex biofilms formed by electrogenic organisms can be engineered to sustain higher current densities (9, 59). Characterizing the fundamental mechanisms and limitations of extracellular electron transfer pathways through current-producing biofilms is essential for the design of electrogenic strains optimized for specific biotechnological applications.

While many respiratory organisms are capable of delivering electrons from the inner membrane to the periplasm, extracellular electron transfer poses the unique physiological challenge of transferring electrons across insulating lipid membranes or outer surface barriers. Different strategies for solving this issue have evolved in Gram-positive bacteria (10), *Archaea* (11), and Gram-negative organisms. The strategy thus far identified in Gram-negative microbes capable of direct extracellular electron transfer utilizes “electron conduits” composed of multiheme cytochromes spanning the outer membrane with the aid of an integral membrane protein (12–14). These conduits conduct electrons to the extracellular space, where additional cytochromes (15, 16), pili (17), and polysaccharides (18) form a conductive extracellular matrix capable of carrying electrons to acceptors many micrometers away.

**FIG 1:**
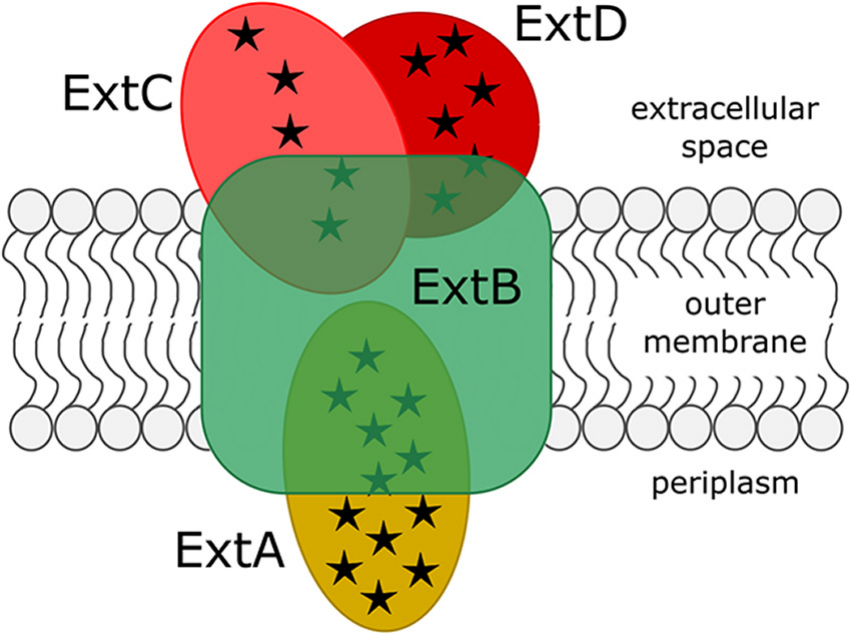
Schematic representation and putative localization of the products of the *extABCD* gene cluster. Putative outer membrane electron conduit made up of ExtA, a dodecaheme periplasmic *c*-type cytochrome, ExtB, an 18-transmembrane-domain integral outer membrane protein, and two extracellular outer membrane *c*-type cytochromes, the pentaheme ExtC and hexaheme ExtD. Numbers of heme-binding sites in each cytochrome are represented by black stars.

The model electroactive organism Geobacter sulfurreducens can couple intracellular oxidation of organic acids to extracellular reduction of many solid electron acceptors, including electrodes, and contains at least five putative outer membrane electron conduits that function in an extracellular-substrate-dependent manner (19). Of the five characterized electron conduits, only ExtABCD (for “extracellular electron transfer”) is involved in electron transfer to electrodes, where ExtA is predicted to be a periplasmic cytochrome, ExtB an integral outer membrane protein, and both ExtC and ExtD extracellular multiheme cytochromes associated with the outer membrane through lipid attachment (Fig. 1) (19, 20). Deleting the four outer membrane electron conduit gene clusters that are unnecessary during electrode reduction (yielding a strain referred to as an *extABCD^+^* strain) does not alter expression of other electron transfer components (i.e., cytochromes, pili, or extracellular polysaccharides) yet results in higher rates of exponential current increase and higher final total current (19) during oxidation of acetate coupled with electron transfer to an electrode. This phenotype is consistent with streamlining theory, which proposes that minimizing cell size and complexity provides an advantage allowing nutrients to be used more efficiently (21).

During growth on electrodes G. sulfurreducens forms ∼50-μm-thick electrode-associated biofilms that reach an upper limit in the rate at which electrons are delivered to an electrode (current density), regardless of electrode geometry or growth conditions (22–24). Intriguingly, this limit is reached even as cells continue to accumulate on electrodes. New growth near the surface pushes older cells farther from the electrode, where they become less active, as indicated by a decrease in the RNA/DNA ratio (23) and cell resolved stable-isotope labeling by NanoSIMS (nanoscale secondary ion mass spectrometry) (25). To explain the increased current density of *extABCD*^+^ biofilms, cells could respire from farther away, altering this anabolic activity stratification pattern, or cells could improve their metabolic rate closer to the electrode. Here, we show that enhanced current production by *extABCD*^+^ biofilms is due to a combination of increased metabolic activity, denser cell packing at the electrode-biofilm interface, and an increase in the apparent diffusion coefficient for electron transfer through the biofilm. These results provide evidence that streamlining the extracellular electron transfer pathway to its essential components accelerates per-cell respiration rates and alters biofilm architecture in a way that improves extracellular electron transfer efficiency. Our results demonstrate the potential to construct electrogenic strains expressing only essential components to produce higher current densities than presently possible.

## RESULTS

### Production of active biomass by *extABCD*^+^ biofilms exceeds that of the wild type.

**FIG 2:**
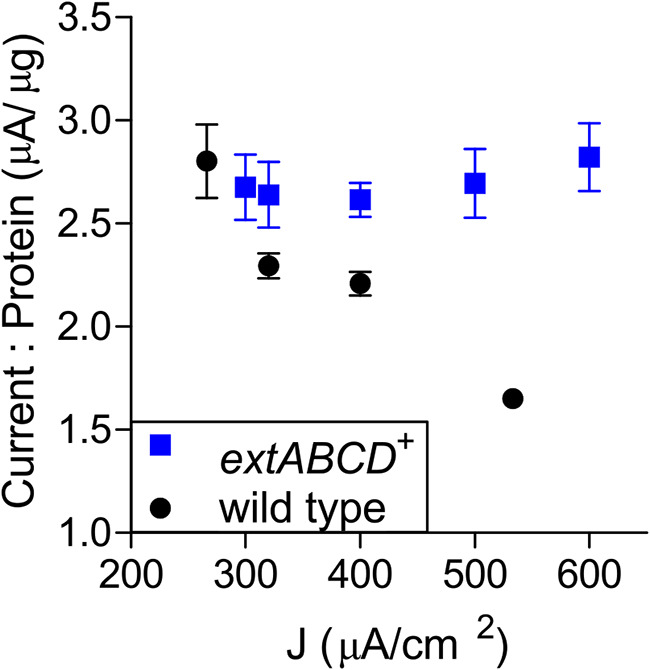
Protein accumulation and current density increase at a constant rate in *extABCD^+^* biofilms, while protein accumulation is not followed by a proportional increase in current density for wild-type biofilms. Total protein content of *extABCD^+^* and wild-type biofilms harvested at increasing current densities. Values for replicate samples are plotted, with error bars representing standard deviations.

Previous studies with wild-type cells demonstrate that an increase in G. sulfurreducens biomass results in a linear increase in current, but only during early exponential phase, when biofilms are <5 μm thick. Beyond this point, biomass accumulation is met with diminishing returns, reflecting the accumulation of cells less and less able to participate in current production (23). When both wild-type and *extABCD*^+^ biofilms were compared at early exponential phase (<300 μA · cm^−1^), each strain showed the typical relationship between current and protein, producing ∼3 μA for every microgram of biomass produced. As previously observed, this ratio decreased steadily to ∼1.6 μA · μg^−1^ in wild-type biofilms as they approached their current density maximum (∼550 μA · cm^−1^), consistent with an increased proportion of noncontributing cells. In contrast, *extABCD*^+^ biofilms maintained a 3-μA · μg^−1^ current-to-protein ratio even as biofilms approached their current density maximum (Fig. 2). This suggests that as the *extABCD*^+^ strain adds new cells to the biofilm, a larger proportion continue to contribute to electrode respiration compared to the wild type.

### Biofilms of the *extABCD*^+^ strain have higher cell density than wild-type biofilms.

**FIG 3:**
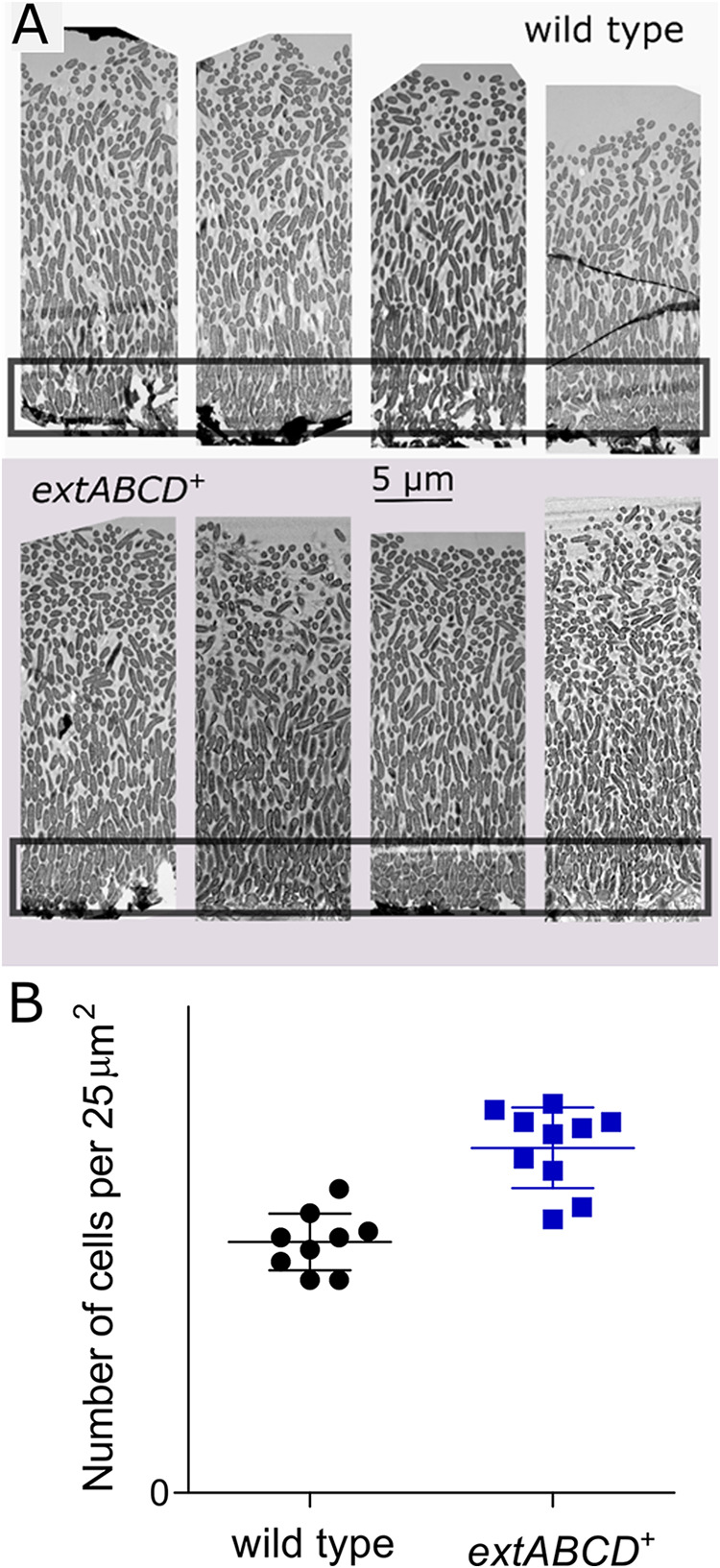
Higher biofilm density in *extABCD*^+^ than wild-type biofilms. (A) Electron microscopy of negatively stained wild-type (top) and *extABCD*^+^ (bottom) G. sulfurreducens biofilms harvested at maximum current production show denser biofilms at the electrode-biofilm interface for *extABCD*^+^ compared to wild type. Representative data from five acquisitions each of two biological replicates per strain. (B) Number of cells per 25 μm^2^ at the electrode-biofilm interface from 10 acquisitions per strain shows denser *extABCD*^+^ biofilms, with 56.8 ± 2.1 cells per 25 μm^2^, versus 41.3 ± 1.5 cells per 25 μm^2^ in wild-type biofilms (*P* < 0.0001).

Because more biomass in *extABCD*^+^ biofilms appeared to participate in current production than in the wild type (Fig. 2), the thickness and structure of biofilms producing maximum current density were determined through electron microscopy. Compared to wild-type biofilms, *extABCD*^+^ biofilms were the same thickness but contained 37.5% ± 0.1% more cells in the first 5 μm from the electrode (representative data are shown in Fig. 3; cell counts from two biological replicates with five acquisitions each from different locations on the biofilm).

### Anabolic activity by individual *extABCD*^+^ cells is higher than that in wild-type cells, while both cell types demonstrate poor growth beyond 10 μm from electrodes.

**FIG 4:**
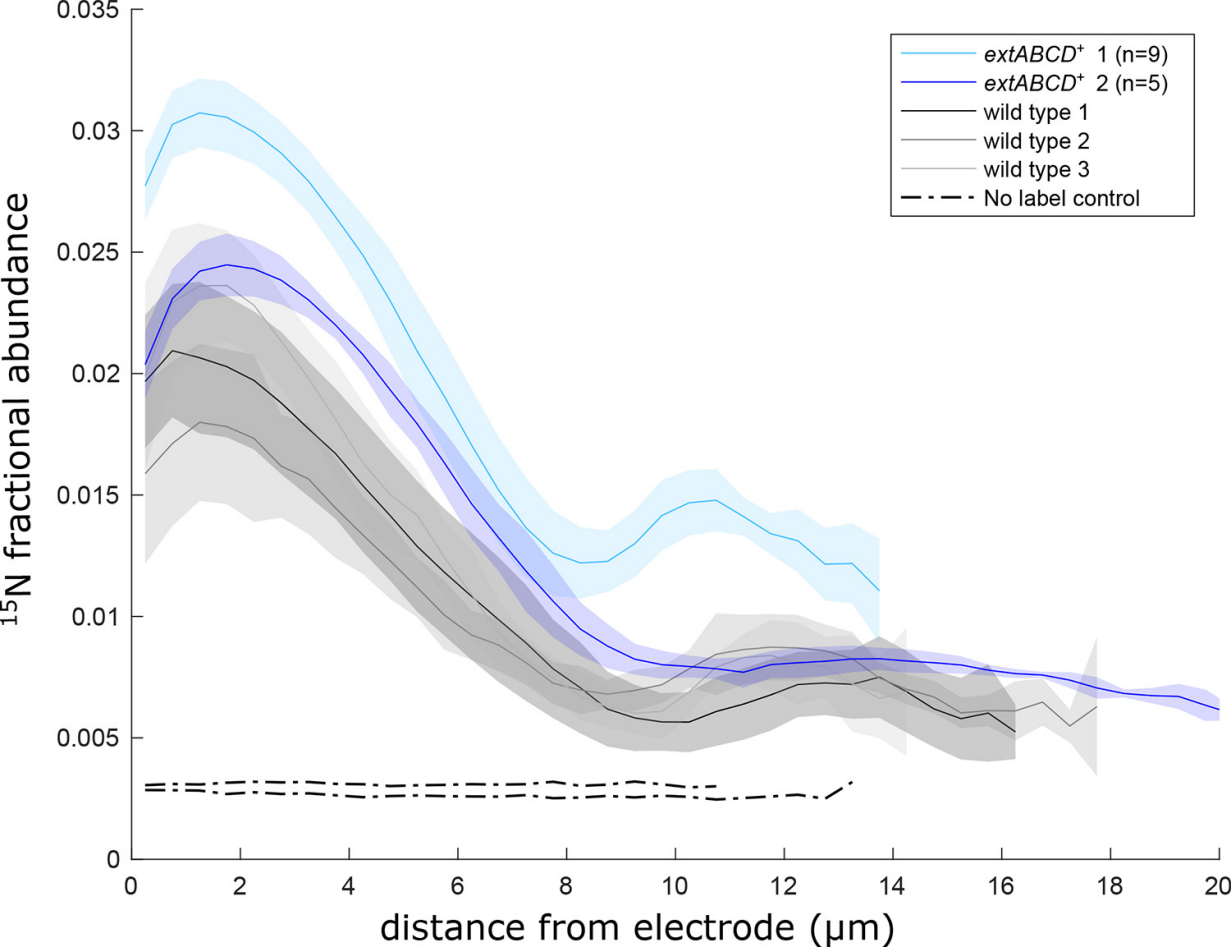
NanoSIMS quantification of cellular anabolic activity by ^15^N incorporation in *extABCD^+^* biofilms compared to wild-type biofilms. *extABCD*^+^ biofilms corresponding to biological replicates are dark and light blue traces; WT replicates are in gray. *extABCD^+^* biofilms producing maximum current were analyzed using NanoSIMS to measure the abundance of ^15^NH_4_ assimilated by cells as a proxy for cell-specific anabolic activity during a 6-h incubation. Data from duplicate *extABCD^+^* biofilms are superimposed on wild-type data (gray) from reference 25, which were acquired in parallel. Solid lines represent average ^15^N fractional abundance in the biofilm at each distance from the electrode calculated from the number of NanoSIMS raster acquisitions shown in parenthesis in the legend with the standard deviation plotted as shaded envelopes surrounding each line. Black dotted lines represent killed controls that were chemically fixed before exposure to ^15^N isotopically labeled medium, confirming that there was no abiotic adsorption of ^15^N isotope.

Our previous stable-isotope incorporation experiments indicated that the highest anabolic activity is located closest to the electrode surface in G. sulfurreducens biofilms and that the activity decays with distance until ∼10 μm, beyond which little growth is observed (25). Using conditions and reagents identical to those used in these experiments with the wild-type strain, *extABCD*^+^ biofilms producing maximum current density were incubated for 6 h (G. sulfurreducens doubling time ≈ 6.2 h) in the presence of isotopically labeled ^15^N, ^13^C, and deuterated water probes and then fixed, stained, and embedded in resin for analysis using NanoSIMS. The anabolically active layer in *extABCD^+^* biofilms was within 5 to 10 μm of the electrode surface, as in wild-type biofilms. However, the peak isotope incorporation within this active zone was 38% higher in *extABCD^+^* biofilms than in wild-type biofilms (Fig. 4).

These data confirmed enhanced anabolic rates, which agrees with the higher growth rate observed for *extABCD*^+^ cells. For example, the fractional abundance of ^15^N added to growth medium was 6%; therefore, unlabeled cells doubling every 6 h should reach a ^15^N fractional abundance value of approximately 3% during the experiment. Peak fractional abundance for the *extABCD^+^* strain was 2.9% ± 0.3%, while it was only 2.1% ± 0.3% for wild-type cells near the anode surface (Fig. 4). From these results, we can infer that during the stable isotope probing experiment, 48% of the cellular biomass near electrodes was new biomass in *extABCD^+^* biofilms, compared to only 35% in wild-type biofilms.

These results corroborate the higher rates of current increase for *extABCD*^+^ at early exponential phase compared to wild type (19) and show that this faster growth continues for cells closest to the electrode even when they are buried within a mature biofilm. While *extABCD*^+^ cells grew faster than wild-type cells, they still showed a “distance penalty,” or decay in growth rate with distance beyond 5 cm from the electrode. A second form of growth rate decline was also observed within the 2 μm closest to the electrode, suggesting acidification nearest to the electrode caused by the higher metabolic rates of *extABCD*^+^. The difference between peak ^15^N fractional abundance and levels near the electrode in wild-type samples was 0.15% but increased to 0.38% for the *extABCD*^+^ strain, suggesting that buffering diffusion limitations previously predicted through modeling (26, 27) might play a more significant role as the anabolic rate increases.

### Electron transfer between cells in *extABCD*^+^ biofilms is faster than that in the wild type.

Because cell density and anabolic activity were higher in *extABCD*^+^ biofilms than in the wild type, we investigated if long-range electron transfer, measured as the rate of diffusion of electrons between cells through the biofilm, was also altered. These experiments were carried out using biofilms grown at +240 mV versus standard hydrogen electrode (SHE) on gold interdigitated array (IDA) electrodes separated by 5-μm gaps (previously used to determine biofilm conductivity for G. sulfurreducens [28]).

**FIG 5:**
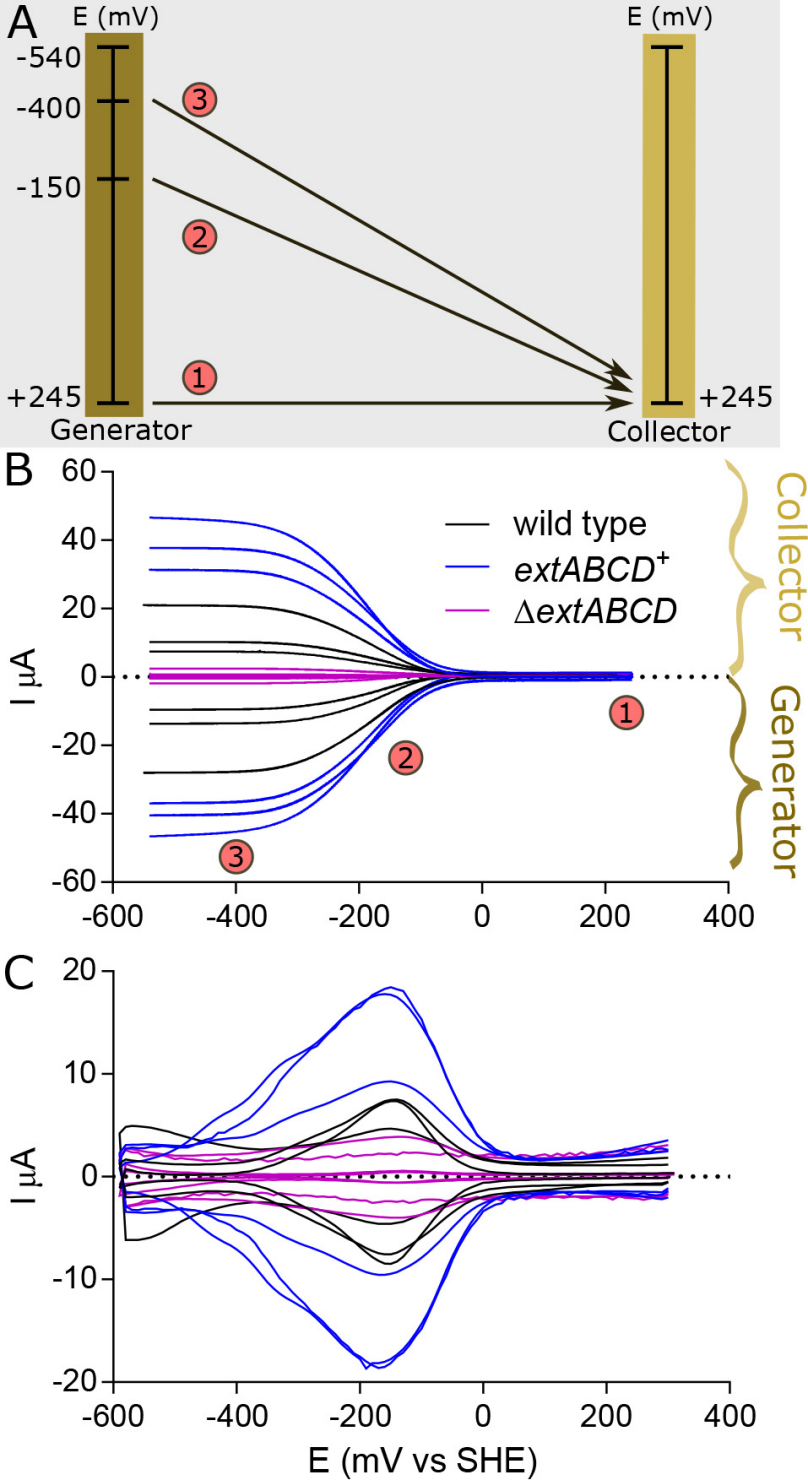
Electrochemical measurements of *extABCD*^+^, Δ*extABCD*, and wild-type biofilms under turnover conditions. (A) Schematic representation of electron flow during generator-collector experiment on IDA electrodes at different potentials within the voltammogram. At stage 1, the equivalent potential of both electrodes results in no electron flow between them. At stage 2, the midpoint potential of redox-active mediators in the conductive biofilm as a whole is reached, and electron flow from reduced to oxidized ends of the biofilm occurs at half-maximal rates. Finally, at stage 3, the maximum current flow between electrodes is reached and is not surpassed regardless of additional increases in the potential difference. (B) Current measured during generator-collector experiments, shown normalized and with each stage from panel A labeled, showing higher rates of current flow through *extABCD*^+^ than wild-type biofilms at all driving forces, and symmetrical curves showing that all current flowing out of the generator electrode is collected at the collector. (C) Square wave voltammetry showing similar difference in higher peak current (*i*_swv_) for *extABCD*^+^ biofilms and wild-type and Δ*extABCD* biofilms.

Current conducted through biofilms was measured by poising one IDA electrode (collector) at +245 mV versus SHE while scanning the other electrode (generator) from +245 mV to −540 mV versus SHE at 1 mV · s^−1^ (Fig. 5A) (29, 30). Such generator-collector measurements create a redox gradient across the 5-μm gap between IDA electrodes to drive electron transfer through the biofilm (monitored as current arriving at the collector) independent of cell metabolism (31). Increasing the magnitude of the redox gradient increases current flux until the potential of the generator electrode is sufficiently reducing and the potential of the collector electrode is sufficiently oxidizing to achieve maximum generator-collector current (*i*_gc,m_), or the maximum rate at which the biofilm can sustain long-distance electron transfer (30, 32). In these experiments, this maximum *i*_gc,m_ was ∼3× higher for *extABCD*^+^ biofilms than for wild-type biofilms (38.23 ± 6.30 μA versus 13.03 ± 7.06 μA; *n* = 3). Negative-control biofilms of the Δ*extABCD* strain reached only one-tenth of wild-type *i*_gc,m_ (Fig. 5B).

A second method able to compare electron transfer through biofilms under turnover conditions is square wave voltammetry, which poises biofilms at a reducing potential for a short period and then discharges them at a slightly higher potential (25 mV in these experiments). Peak current in square wave voltammetry analysis (*i*_swv_) from these was reached at a redox potential similar to the midpoint potential of generator-collector measurements (square wave peak of −159.3 ± 5.9 mV versus a generator-collector midpoint of −154.0 ± 8.0 mV), suggesting that the same electron transfer mediators were responsible for both types of measurements. The magnitude of the peak current in square wave analysis (*i*_swv_) was higher for *extABCD*^+^ than for wild-type biofilms, 14.5 ± 4.5 μA versus 7.9 ± 0.2 μA, respectively (Fig. 5C), consistent with a higher maximum rate of redox-driven long-distance electron transfer for *extABCD*^+^ biofilms. Δ*extABCD* biofilms were used as negative control, but *i*_swv_ was not significantly different from background current for this strain.

### *extABCD*^+^ biofilms have a higher apparent electron diffusion coefficient.

Biofilm conductivity (σ) describes the rate at which long-distance electron transfer can occur via a gradient-driven process (physical diffusion of charge carriers or redox conduction via electron transfer reactions among bound charge carriers such as multiheme *c*-type cytochromes) and is a function of both the apparent diffusion coefficient (D) and the effective concentration of charge carriers (*C*_T_), described by equations 7 and 10 (29, 30, 33). Whereas generator-collector experiments reflect the (quasi)steady state of the apparent diffusion coefficient multiplied by the concentration of charge carriers in the biofilm (equation 5), transient current generated during square wave voltammetry is dependent on the square root of the apparent diffusion coefficient multiplied by the concentration of the charge carriers in the biofilm (equation 1). Because experiments were performed on identical biofilms, the quotient of *i*_swv_/*i*_gc,m_ recorded for the same biofilm provides a means to solve for *D* and *C*_T_ separately (see Materials and Methods) (30).

**FIG 6:**
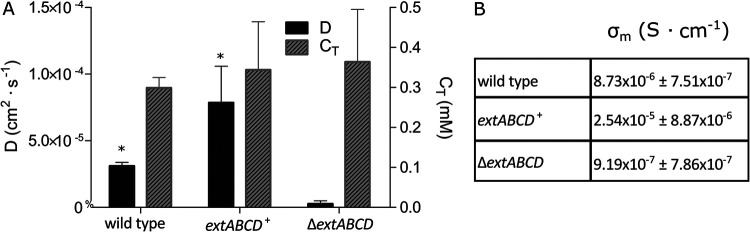
Charge transfer parameters of wild-type, *extABCD*^+^, and Δ*extABCD* biofilms. (A) Charge carrier concentration (*C*_T_) and diffusion coefficient (*D*) (*n* = 3; *, *P* = 0.0379). (B) Conductivity (σ_m_) of electron transport through biofilm matrix values calculated from *i*_gc_ and *i*_swv_.

According to these measurements, the apparent diffusion rate, and not the concentration of carriers within biofilms, was significantly different between wild-type and *extABCD*^+^ biofilms (3.11 × 10^−5^ ± 2.7 × 10^−6^ cm^2^ · s^−1^ versus 7.85 × 10^−5^ ± 2.74 × 10^−5^ cm^2^ · s^−1^; *n* = 3; *P* = 0.0379). According to these model-based calculations, in which the biofilm is assumed to be homogeneous, electron transfer reactions occur ∼2.5× faster among bound charge carriers in *extABCD*^+^ than in wild-type biofilms (Fig. 6A). Moreover, diffusion of electrons within *extABCD*^+^ biofilms was ∼30× faster than in biofilms of the deletion mutant Δ*extABCD*, suggesting that the ExtABCD conduit not only may play a role in the process of electron transfer across the outer membrane but also may contribute to between-cell electron transfer networks within biofilms. For example, as cells were more closely packed in *extABCD*^+^ biofilms, increased rates of between-cell electron transfer could be possible without increasing the overall number of charge carriers known to be outside the cell, such as OmcZ.

### Overexpression of *extABCD* or individual subunits is detrimental to electrode reduction.

Since *extABCD* is the only electron transfer conduit in G. sulfurreducens we have found to be linked to electrode respiration, and expression of this gene cluster is relatively low during electrode growth (e.g., at a level equivalent to only 5% of periplasmic cytochromes such as that encoded by *ppcA*), increasing its expression level could increase performance. Beginning with a Δ5 strain, which has all five characterized electron conduit gene clusters deleted (19), strains were constructed with either an empty pGeo2 vector, an *extABCD* transcriptional unit under the control of either the *extA* promoter (p-P*_extA_-extABCD*) or a constitutive G. sulfurreducens promoter supporting 9-fold-higher expression levels than *extA* (p-P*_acpP_-extABCD*) (19, 34, 35).

**FIG 7:**
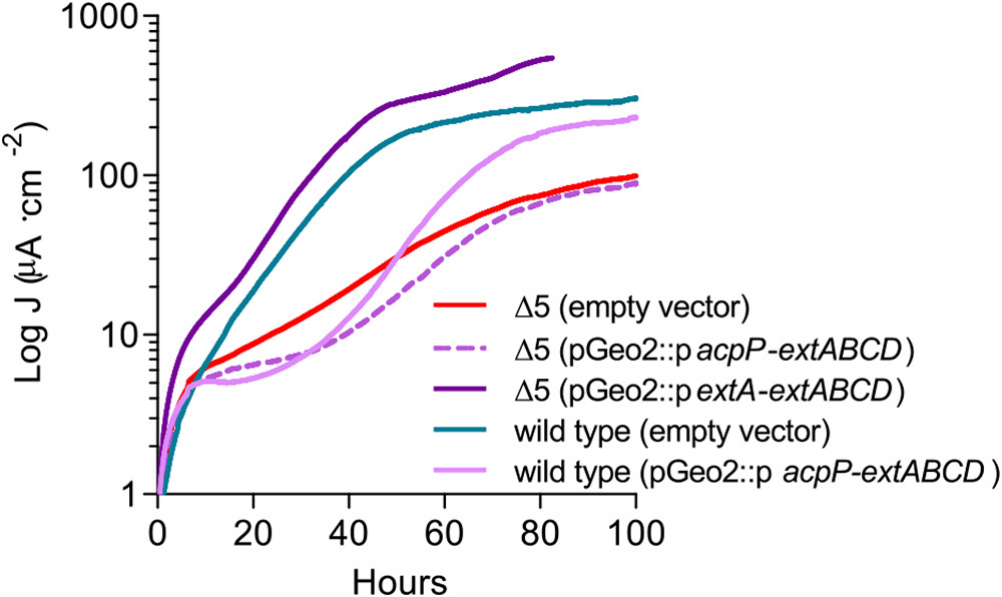
Overexpression of *extABCD* does not rescue Δ5 electrode reduction activity and causes defects in the wild type. Current production of wild-type and Δ5 strains carrying either empty pGeo2 plasmids, p-P*acpP*-*extABCD*, or p-P*extA-extABCD* and grown using poised electrodes as the sole terminal electron acceptor. Curves are representative (*n* = 4).

Only the native promoter construct, p-P*_extA_-extABCD*, could rescue the Δ5 strain phenotype. Expressing *extABCD* under the control of a stronger P*_acpP_* promoter resulted in no increase of current production by the Δ5 strain (Fig. 7). In order to further test the hypothesis that there was a detrimental effect of overexpressing *extABCD* under electrode-reducing conditions, a wild-type strain carrying p-P*acpP-extABCD* was also analyzed. This strain, containing both a wild-type genomic copy and a plasmid-borne copy of *extABCD*, demonstrated a long lag and reduced final current density compared to the wild type carrying the empty vector (Fig. 7). These results suggest that increasing expression of *extABCD* is actually disadvantageous during electron transfer to electrodes.

### Single-gene deletions of *extB*, *extC*, and *extD* produce stronger defects in electrode reduction than the deletion of *extA*.

**FIG 8:**
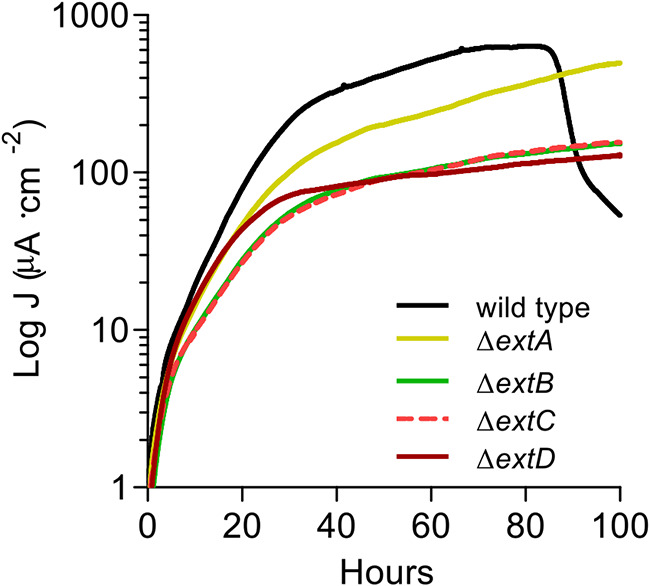
Deletion of *extB*, *extC*, or *extD* results in severe deficiency in electrode reduction. Single-gene-deletion mutants of *extA*, *extB*, *extC*, and *extD* were grown using graphite electrodes poised at +240 mV versus SHE as the sole terminal electron acceptor. Representative curves (*n* = 4) of current production over time are shown.

In order to determine if the entire *extABCD* cluster was required for electron transfer to electrodes, single markerless deletions of each gene were constructed and analyzed for their ability to reduce electrodes poised at +240 mV versus SHE. Individual deletions of *extB*, *extC*, and *extD* were as detrimental to current production as deletion of the full *extABCD* gene cluster (Fig. 1), with final current density never surpassing 100 μA · cm^−2^ in any of these mutants (Fig. 8) (*n* = 4). Surprisingly, deletion of *extA* alone resulted in a less pronounced defect, with doubling time slowed to ∼8 h and final current density eventually reaching ∼490 μA · cm^−2^ after 100 h, a level near that of the wild type (Fig. 8) (*n* = 4). These results indicate that although *extA* mRNA levels are often higher than *extBCD* during electrode reduction (19, 35), other periplasmic cytochromes may be able to compensate, and lack of *extBCD* encoding the putative outer membrane integral protein and extracellular cytochromes are primarily responsible for the defective phenotype of Δ*extABCD*.

## DISCUSSION

Enhancing the current output of electrogenic biofilms by at least an order of magnitude is essential to biotechnological systems that rely on microbial current production (3, 8). If cells can grow only as a monolayer, total current on an electrode can be improved by increasing electrode surface area to allow more cells to colonize or increasing the rate at which each cell can deliver electrons to a surface. If cells can form a conductive biofilm, altering the conductivity of the biofilm can affect how far away from an electrode cells can actively respire, increasing the number of total cells participating in current production. As a step toward improving electrogenic biofilm current output, we sought to determine which of these factors contributed to enhanced current production by the *extABCD*^+^ strain (a G. sulfurreducens mutant lacking four outer membrane electron conduit gene clusters involved in metal oxide reduction but containing *extABCD* [19]). Strains were analyzed in depth using electron microscopy, biomass measurements, stable isotope probing (NanoSIMS), conductivity measurements, and electrochemical analysis. Our results show that the main differences between wild-type and *extABCD^+^* biofilms are that (i) *extABCD*^+^ biofilms are able to form more densely packed biofilms near the electrode-biofilm interface, increasing the number of active current-producing cells, (ii) each cell in this crucial active zone is capable of higher per-cell anabolic activity, and (iii) measurements of electron transfer parameters suggest that this effect is enabled by faster diffusion of electrons within the *extABCD*^+^ biofilms.

When biofilms use electrodes as electron acceptors, the metabolic state of cells is heterogeneous, or metabolically stratified, due to cells closest to the electrode experiencing the highest redox potential and overactive cells possibly poisoning themselves due to acidification (25, 26). In all electrode-based observations, isotope incorporation of labeled carbon and nitrogen within current-producing biofilms is highest within the first 10 μm (25) and the redox state of cytochromes is most oxidized at the electrode-biofilm interface, suggesting that the environment closest to the electrode remains favorable even when buried beneath tens of micrometers of biomass (36–40). Our results showing higher current densities in *extABCD*^+^ biofilms as a result of more densely packed cells and higher ^15^N incorporation rates within a similar 0- to 10-μm active zone shows that this steep stratification favoring the region near the electrode also occurs in *extABCD*^+^ biofilms. However, our results show evidence for a new penalty at the electrode-biofilm interface in *extABCD*^+^ biofilms compared to wild-type biofilms, consistent with previously modeled effects of proton accumulation due to the increased anabolic rate beginning to limit current production (26, 27).

Because long-distance electron transfer supports growth of cells not in contact with the electrode, higher rates of activity (Fig. 4) should require an increase in the electron transfer parameters. In agreement with this, model-based calculations (where the biofilm is assumed to be homogeneous) based on two separate measurements of electron transfer are consistent with an increase in the rate of electron diffusion (*D*), but not the overall concentration of charge-carrying mediators (*C*_T_), in *extABCD*^+^ biofilms. While measurements point to faster electron transfer within *extABCD*^+^ biofilms, the physiological origin of this faster diffusion coefficient is yet to be determined.

The absence of unnecessary outer membrane cytochrome conduits could allow faster electron diffusion due to many factors, with the main hypotheses being the following.

(i) Unnecessary outer membrane cytochromes act as dead-end electron sinks, extending the length of an electron’s random walk and slowing the rate of electron diffusion in the wild type, as suggested by previous electrochemical surface plasmon resonance measurements (41).

(ii) The absence of bulky outer membrane proteins enables closer packing of cells and thus contact between electron carriers. While cell-cell electron transfer is attributed to cytochromes secreted between the cells such as OmcZ (15, 42, 43), ExtABCD complexes could make contact directly, similar to electron transfer along Mtr complexes produced by Shewanella oneidensis (44). The counterintuitive relationship between more closely packed cells without an accompanying increase in electron carrier concentration may be due to the absence of the OmbB-OmaB-OmcB and OmbC-OmaC-OmcC outer membrane complexes in the *extABCD*^+^ strain, since these represent some of the most abundant cell-associated multiheme *c*-type cytochromes in wild-type G. sulfurreducens (14, 19, 45). The rate of electron diffusion, or the rate at which electrons traveled between electrodes, increased by ∼2.5-fold in the *extABCD*^+^ strain compared to the wild type, which is an expected effect of closer cell-cell contact. This increased conductivity is more than enough to support the higher anabolic activity of *extABCD*^+^ measured by NanoSIMS. The fact that long-range electron transfer decreased by ∼30-fold in Δ*extABCD* biofilms provides evidence that cytochromes on the outer surface might also participate in cell-cell electron transfer, but the poor growth of this mutant on electrodes makes direct comparisons difficult. Additional effects of closer cell packing on the arrangement of the extracellular polymer matrix cannot be discarded with the available data.

(iii) A final possibility is that the absence of unnecessary outer membrane electron conduits facilitates direct interaction of ExtABCD with the electrode to alleviate a bottleneck within the biofilm-to-electrode electron transfer process. This would not be captured in models used to calculate diffusion coefficients, which assume infinitely fast heterogeneous electron transfer from the biofilm to the electrode. These hypotheses can be tested by biochemical and electrochemical characterization of ExtABCD and its partners.

It is worth noting that values for electron transfer parameters presented here, a *D* value of 3.11 × 10^−5^ cm^2^ · s^−1^ and a *C*_T_ value of 0.3 mM, are significantly higher and lower, respectively, than those previously reported for G. sulfurreducens wild-type biofilms (46). Since these parameters cannot be measured directly, all methods require estimation of the amount of electron transfer mediators involved, usually by integration of voltammetry and estimation of the biofilm thickness (46). In our experiments, the diffusion coefficient of long-range electron transfer and the concentration of charge carriers in the biofilm were determined by two unambiguous experimental measurements, the expected generator-collector current (*i*_gc_) and *i*_swv_. The maximum conductivity calculated from these values for G. sulfurreducens wild-type biofilms (8.7 × 10^−6^ S · cm^−1^ [Fig. 6B], based on equation 10, which we derive here) closely matches σ_m_ determined experimentally by electrochemical gating measurements (5.5 × 10^−6^ S · cm^−1^) (28). Future calculations of electron transfer parameters will need to confront in their models the assumption that conductive biofilms are homogeneous, as isotopic label incorporation shows that both *extABCD*^+^ (Fig. 4) and wild-type (25) biofilms are stratified into layers with decreasing anabolic activity with increased distance from the electrode, and electron microscopy shows a gradient of decreasing cell density with distance. Rather than attempt to determine unequivocal values for the diffusion coefficient, concentration of charge carriers, or total conductivity of the biofilm, our goal was to directly compare charge transfer parameters under identical conditions in biofilms formed by three strains that differ only in outer membrane cytochrome content.

Overexpression of critical pathway components is a standard technique in traditional metabolic engineering, but as seen for other integral membrane proteins (47, 48), overexpression of *extABCD* did not produce the desired effect. In the particular case of *extABCD*, toxicity is not probable, since defective phenotypes were evident only when extracellular terminal electron acceptors were used as substrates and not during respiration of fumarate (Fig. 7) (19). Higher expression of *extABCD* may lead to misfolding of independent subunits, and it has been shown that the cytochrome maturation pathway is a bottleneck for the extracellular electron transfer pathway (49). It is also possible that interactions between ExtABCD and its periplasmic/extracellular partners are disrupted with higher ExtABCD abundance, affecting the functionality of the whole extracellular electron transport pathway. For example, recent models predict that different nanowires composed of polymerized cytochromes are expressed for use within the biofilm (50). Efficient electron transfer from the outer membrane to these nanowires in the extracellular matrix may require a specific stoichiometry of outer membrane electron conduits to partnering nanowires. Additional biochemical characterization of ExtABCD could address these questions and aid identification of its extracellular partners.

Biofilms are notoriously complex environments containing multiple microhabitats and limitations, and conductive biofilms on electrodes are no different. Together, our data show that deletion of four unnecessary gene clusters produced a G. sulfurreducens strain able to grow in a denser biofilm morphology, concentrating cells with higher anabolic activity near the electrode, where they were supported by apparent faster long-range electron transfer. This enhanced overall rate of current production was a result of addressing only one component of a much larger extracellular electron transfer pathway that includes inner membrane, periplasm, and extracellular matrix proteins. This work provides a guide for engineering these additional cellular compartments, showing not only possible positive outcomes but also potential pitfalls of overexpression of multiheme cytochromes in a tightly controlled system. With fundamental characterization of the complete extracellular electron transfer pathway (15, 16), electrode-optimized strains appear possible, which will ultimately increase current output at the core of multiple microbial electrochemistry technologies.

## MATERIALS AND METHODS

### Cell growth and electrode reduction assays.

Geobacter sulfurreducens strain PCA colonies picked from freezer stocks were used to start all cultures in vitamin-free freshwater liquid medium (0.38 g/liter KCl, 0.2 g/liter NH_4_Cl, 0.069 g/liter NaH_2_PO_4_ · H_2_O, 0.04 g/liter CaCl_2_ · 2H_2_O, 0.2 g/liter MgSO_4_ · 7H_2_O, 1% [vol/vol] trace mineral mix [pH 6.8] buffered with 2 g/liter NaHCO_3_ and flushed with 80:20 N_2_-CO_2_ gas mix) with 20 mM acetate and 40 mM fumarate. For electrode reduction assays, three-electrode sterile conical reactors (BASi, USA) with custom tops (see https://bondlab.umn.edu/research-projects for CAD drawings) containing 15 ml of anaerobic freshwater liquid medium with 40 mM acetate and 50 mM NaCl were flushed with N_2_-CO_2_ gas mix until O_2_ levels reached less than 2 ppm (∼15 min). G. sulfurreducens cultures reaching electron acceptor limitation with an optical density at 600 nm (OD_600_) between 0.48 and 0.52 were used to inoculate electrochemical cells in a 25% (vol/vol) ratio. Graphite working electrodes were poised at +0.24 V versus SHE, and average current density was recorded every 10 s. All strains and plasmids used in this study can be found in Table 1.

**TABLE 1:** G. sulfurreducens strains and plasmids used in this study

Strain or plasmid	Description	Reference
Strains		
DB2041	ΔGSU2645 (Δ*extA*)	This study
DB2040	ΔGSU2644 (Δ*extB*)	This study
DB2039	ΔGSU2643 (Δ*extC*)	This study
DB2038	ΔGSU2642 (Δ*extD*)	This study
DB1280	ΔGSU2645-42 (Δ*extABCD*)	58
DB1290	ΔGSU2731-39 ΔGSU2940-36 ΔGSU2724-26 (*extABCD*^+^)	19
DB1493	ΔGSU2731-39 ΔGSU2645-42 ΔGSU2726-24 ΔGSU2940-36 (Δ5)	19

Plasmids		
pRK-Geo2		58
pRK-Geo2i		58
p-P*extA*-*extABCD*		19
p-P*acpP*-*extABCD*		This study

### Genetic deletion and complementation strategy.

Each gene within the *extABCD* gene cluster was deleted individually. Deletion mutant construction was achieved by using ∼750 bp flanking the target region to induce homologous recombination via the suicide vector pK18*mobsacB* (51) as previously described (34). Briefly, after selection for kanamycin resistance, indicating a genomic insertion of the suicide vector, 10 colonies were exposed to 10% sucrose to induce a second round of homologous recombination, resulting in either the wild-type or deletion allele. Successful deletion mutants were confirmed using kanamycin sensitivity, PCR amplification with flanking primers, and sequencing of target regions. Constitutive complementation strains were constructed using the G. sulfurreducens expression vector pRK2-Geo2 as the backbone and using either its native P*_acpP_* promoter or the P*_extA_* promoter. Inducible complementation strains were constructed using pRK2-Geo2i as the backbone, which controls the expression of P*_acpP_* via VanR-dependent induction. Primers used for this study can be found in Table 2.

**TABLE 2:** Primers used in this study

Purpose and name	Sequence	
Construction of deletion vectors^*a*^		
GSU2642 U1 XbaI	ACGTCG TCTAGA CCT CAC CTA TGA CAG CCG GTT C	
GSU2642 U2	GCAGGCGGCGTCAACGAAC CCT CTT CAT TGC CAG CGT GCT	
GSU2642 L1	AGCACGCTGGCAATGAAGAGG GTT CGT TGA CGC CTG C	
GSU2642 L2 HindIII	ACGTCG AAGCTT CGC GAA CTG CGA TGG AAA CGT AG	
GSU2643 U1 XbaI	ACGTCG TCTAGA CGG TAT CTC GAT GTT CGC TCA TTC G	
GSU2643 U2	CCGATCCGTGAAATCACCGTTAACC GGC GAG AAG CAT GCA CCC	
GSU2643 L1	GGGTGCATGCTTCTCGCC GGT TAA CGG TGA TTT CAC GGA TCG G	
GSU2643 L2 HindIII	ACGTCG AAGCTT GAC AGA GAA CGC AGT CGC GTA C	
GSU2644 U1 XbaI	ACGTCG TCTAGA CTT CAC CTG TCA AGG CTG TCA C	
GSU2644 U2	TCCATCACGCTCTTACCTGCG GGT CAT CCA GGA ACG C	
GSU2644 L1	GCGTTCCTGGTGGATGACC CGC AGG TAA GAG CGT GAT GGA	
GSU2644 L2 HindIII	ACGTCG AAGCTT GAC AGA CCT TGC ACT GGT TGA GG	
GSU2645 U1 XbaI	ACGTCG TCTAGA CTT CAA TGT GAG CGA TGG TCA CC	
GSU2645 U2	CATCACAACGGACTGTCAGCG GGC AAC CAT CGC CAC CAA G	
GSU2645 L1	CTTGGTGGCGATGGTTGCC CGC TGA CAG TCC GTT GTG ATG	
GSU2645 L2 HindIII	ACGTCG AAGCTT CGG AAC GGT CGT TGA GAT AGT C	
Confirmation of gene deletion		
Δ*extD* (ΔGSU2642)	GAC GCT CAA TCT TCT GAC GGG C	
	CTG TCG GCA GTG CGC TAC TTG	
Δ*extC* (ΔGSU2643)	CGG AGC GAG GAG CTT CTG G	
	GGC GTC AAC GAA CGA TTG TCG	
Δ*extB* (ΔGSU2644)	CTC CGC GTT TCA GGA CAT CAA G	
	AGC ACC GAG CAG GTT GGT T	
Δ*extA* (ΔGSU2645)	GTG GCG TGT ACG GCG ATT G	
	CGG TCA CCG AGT ACC GTC TG	
Construction of complementation plasmids		
p-P*acpP*-*extABCD* (NdeI and SacI)	ACGTCG CATATG GTT GCC CTG TTC GGA TG	
	ACGTCG GAGCTC TCA ACG AAC GAT TGT CGG ATG ACA G	
p-P*extA*-*extABCD*
GSU2645 PextA U1 AscI	ACGTCG GGCGCGCC CGG CCA TTT CAT TGC TTG ACA GG	
GSU2645 P*extA* U2	CAATGCATCCCCCTCCTCGTG TCA GCG CTG ACG AAC CGG	
GSU2644 L1	CCGGTTCGTCAGCGCTGA CAC GAG GAG GGG GAT GCA TTG	
GSU2642 L2 BglII	ACGTCG AGATCT GCA GGC GGC GTC AAC GAA C	

a U1-U2 and L1-L2 primers were used to amplify upstream and downstream ∼750-bp flanking regions of target gene. Overlapping PCR was used to combine both products into the insert, which was then ligated into the multiple-cloning site of pK18*mobsacB* using the indicated restriction enzymes.

### Current-to-protein ratios.

Eight wild-type biofilms and 10 *extABCD*^+^ biofilms were harvested at increasing current densities, resulting in two biological replicate samples for each current density sampled, with the *extABCD*^+^ sample set having an additional current density sample because this strain reaches current densities above the wild-type current density limit. Planktonic cells around each biofilm were removed by submerging graphite flags in 1 ml of freshwater liquid medium. Biofilms still attached to graphite flags were then incubated in 1 ml of 0.2 N NaOH at room temperature for 1 h and frozen at −4°C for at least 24 h. Protein concentration was measured using the Pierce bicinchoninic acid (BCA) protein assay kit (Thermo Scientific) with bovine serum albumin (BSA) standards prepared in 0.2 NaOH and treated in parallel to biofilm samples. The absorbance of each standard and sample was measured in triplicate at a wavelength of 562 nm. Blank-sample absorbance was subtracted from all measurements, and concentration of samples was determined from a BSA standard curve.

### Stable isotope probing.

The protocol for stable isotope probing was followed as previously described (25). Briefly, biofilms at the current plateau stage (∼80 h) were labeled by carefully exchanging medium for medium identical in chemical composition to normal growth medium but with increases in the final heavy isotope of ^15^N to 6 atom% in ammonium, ^13^C to 6 atom% in both acetate carbons, and ^2^H to 2 atom% in the water. Enriched isotopic chemicals were purchased from Cambridge Isotope (^15^NH_4_Cl [NLM-467]) and Sigma (D_2_O [151882] and ^13^CH_3_^13^CO_2_Na [282014]). Biofilms were incubated for 6 h, corresponding to one G. sulfurreducens doubling, under regular electrode reduction conditions before biofilms were harvested for staining and embedding.

### Biofilm fixation and embedding.

A previously described protocol for biofilm fixation and embedding was followed (25). Briefly, biofilms attached to graphite electrodes were harvested from each reactor and were fixed at room temperature for 1 h (2% glutaraldehyde, 50 mM HEPES [pH 6.8]) and rinsed twice (50 mM HEPES [pH 7]) before negative staining in 1% OsO_4_, 50 mM HEPES (pH 7) for 2 h and 1% uranyl-acetate for 1 h. Samples were dehydrated with sequential 10-min incubations in 25, 50, 75, and 100% ethanol (EtOH) and embedded in LR White resin (Sigma-Aldrich; catalyzed with benzoyl peroxide).

### Sample preparation for NanoSIMS.

A previously described sample preparation protocol was followed (25). Briefly, slices of resin-embedded biofilms still attached to electrodes were cut perpendicular to the largest face of the electrode using a microtome and glass knife. Thin sections between 200 and 500 nm composed of electrode-attached biofilm were cut for NanoSIMS analysis with a diamond knife. Floated sections were collected on glow-discharged 7- by 7-mm silicon wafers (Active Business). Biofilm sections on silicon wafers were coated with 40-nm gold using a Cressington sputter coater.

### Electron microscopy.

A subset of biofilm sections were imaged with transmission and scanning electron microscopy (TEM and SEM). For TEM, 100-nm sections were cut using a diamond knife, collected on copper TEM grids, and imaged on an FEI Tecnai (T12) microscope operated at 120 keV. Sections collected on silicon wafers (described above) were imaged on a Merlin Compact scanning electron microscope (Zeiss).

### NanoSIMS data acquisition.

A previously described protocol for data acquisition was followed (25). Briefly, isotope enrichment data were collected on a Cameca NanoSIMS 50L housed in the Center for Microanalysis at the California Institute of Technology. Six masses were collected corresponding to the ^1^H^−^, ^2^H^−^, ^12^C^−^, ^13^C^−^, ^14^N^12^C^−^, and ^15^N^12^C^−^ ions, for the determination of ^2^H/^1^H, ^13^C/^12^C, and ^15^N/^14^N ratios, respectively, using a tuning similar to that described by Kopf et al. (52).

### Data processing.

NanoSIMS.im data files were initially processed using the Look@NanoSIMS Matlab graphical user interface (GUI) (53) to align planes and export raw data. All subsequent data processing and analysis were done in Matlab. Regions of acquisitions that contained *Geobacter* biofilm were outlined on the ^14^N^12^C^−^ mass image, where the surface of the electrode was traced by hand, and each pixel of *Geobacter* biofilm was assigned a minimum distance to the electrode surface by calculating the pairwise distance between each pixel in the biofilm and the electrode surface. Biofilm pixels were assigned to bins of 0.5-μm increments from the anode surface, and the ^15^N^12^C^−^ and ^14^N^12^C^−^ counts were pooled for each distance bin to calculate the fractional abundance of the heavy isotopes: ^15^F = ^15^N^12^C^−^/(^15^N^12^C^−^ + ^14^N^12^C^−^). Pixels with low ^14^N^12^C counts corresponding to the epoxy resin between cells were omitted, as was the bin furthest from the electrode when it contained very few pixels.

### Electron transfer parameter characterization.

G. sulfurreducens biofilms were grown on gold interdigitated array (IDA) electrodes with 10-μm-wide electrodes separated by 5-μm gaps serving as working electrodes with graphite rods as counter-electrodes and Ag/AgCl reference electrodes as previously described (32). Jacketed microbial electrochemical reactors maintained at 30°C were used for these measurements with 150 ml of freshwater liquid electrode medium, described above. Electrochemical measurements were performed once biofilms reached a current plateau (∼80 h) under turnover conditions in the presence of acetate as the electron donor. Square wave voltammetry was performed with both IDA electrodes shorted as a single working electrode sweeping from 300 to −590 mV, versus SHE, with an amplitude of 25 mV, a period of 70 ms, increment of 10 mV, and sampling width of 35 ms. Generator-collector experiments were performed under turnover conditions by poising one IDA electrode at +240 mV versus SHE, scanning the other from +240 to −550 mV versus SHE at 1 mV · s^−1^, and recording current produced at each electrode to assess biofilm conductivity. Assuming that electroactive biofilms are redox conductors in which long-distance electron transfer results from sequential electron transfer reactions between neighboring reduced and oxidized redox sites in a bucket brigade manner (28, 32, 33), the effective concentration (*C*_T_) of electron transfer mediators, taken here to be heme cofactors of membrane-associated and extracellular cytochromes involved in long-distance electron transfer through the biofilm (29, 54, 55), and the apparent diffusion coefficient (*D*), a measure of how rapidly electron transfer occurs for a given *C*_T_ and redox gradient (below) following Fick’s first law (33, 56), were calculated as previously described (30).

For square wave voltammetry (SWV) performed on IDA-grown biofilms, the expected peak current (*i*_swv_) can ideally be expressed as:

(1) $$i_{\mathrm{swv}}=\frac{\phi \mathrm{nFA}\sqrt{D}C_{T}}{\sqrt{\pi }\sqrt{t_{p}}}$$

where the constant ϕ of 0.5879 and the pulse width (*t*_p_) of 0.07 s are experimental parameters used for SWV, a value for *A* of 0.039 cm^2^ is the electrode surface area, a value for *F* of 96,485 C · mol^−1^ is the Faraday constant, and an *n* value of 1 is the number of electrons per change in oxidation state of electron transfer mediators that occurs during long-distance extracellular electron transfer (taken as individual hemes).

The expected generator-collector current (*i*_gc_) can be ideally expressed as:

(2) $$i_{\mathrm{gc}}=\mathrm{nFDS}C_{T}(P_{g}-P_{c})$$

(3) $$P_{g}=\frac{1}{1+e^{\left[\frac{\mathrm{nF}(E_{g}-E^{{}^{\circ}\prime })}{\mathrm{RT}}\right]}}$$

(4) $$P_{c}=\frac{1}{1+e^{\left[\frac{\mathrm{nF}(E_{c}-E^{{}^{\circ}\prime })}{\mathrm{RT}}\right]}}$$

where *E*_g_ and *E*_c_ are the potentials applied to the generator and collector electrodes relative to the formal potential of the electron transfer mediators, E°′ (33), and a value for *S* of 14.5 cm is a constant based on the IDA geometry (32). Here, the term in parentheses in equation 2 describes the resulting redox gradient that drives electron transfer through an electroactive biofilm from the generator to the collector electrodes, where *P*_g_ is the fraction of electron transfer mediators maintained in the reduced state at the biofilm-generator interface in response to *E*_g_, and *P*_c_ is the fraction of electron transfer mediators maintained in the reduced state at the biofilm-collector interface in response to *E*_c_, where *P*_g_ and *P*_c_ are calculated from the Nernst equation. In the limit that *E*_g_ << *E*°′, *E*_c_ << *E*°′, *P*_g_ is equal to 1 (all electron transfer mediators are maintained in the reduced state at the biofilm-generator interface), and *P*_c_ is equal to 0 (all electron transfer mediators are maintained in the oxidized state at the biofilm-generator interface), the largest possible redox gradient is generated through the biofilm between the generator and collector electrodes. This results in the maximum generator-collector current, *i*_gc,m_, which can ideally be expressed as:

(5) $$i_{\mathrm{gc,m}}=\mathrm{nFDS}C_{T}$$

Implicit in this model is the assumption that the rate of electron transfer from the generator electrode into the biofilm as well as the rate of electron transfer from the biofilm into the collector electrode are not rate limiting.

Combining equations 1 and 5 provides a solution for *D*:

(6) $$D={\left(\frac{\phi A}{\sqrt{\pi }\sqrt{t_{p}}S\frac{i_{\mathrm{swv}}}{i_{\mathrm{gc,m}}}}\right)}^{2}$$

that can be determined from the ratio of two experimentally measured currents (*i*_swv_/*i*_gc,m_). With *D* in hand, either of the two first expressions can be used to solve for *C*_T_ (57).

Following equation 2, conductivity (σ), which characterizes the degree to which a biofilm conducts electrical current by redox conduction-based extracellular electron transfer, can ideally be expressed as:

(7) $$\sigma =\frac{i_{\mathrm{gc}}}{\mathrm{VS}}=\frac{n^{2}F^{2}DC_{T}e^{\left[\frac{\mathrm{nF}}{\mathrm{RT}}(E_{G}-E{}^{\circ}\prime )\right]}}{\mathrm{RT}(1+2e^{\left[\frac{\mathrm{nF}}{\mathrm{RT}}(E_{G}-E{}^{\circ}\prime )\right]}+e^{\left[2\frac{\mathrm{nF}}{\mathrm{RT}}(E_{G}-E{}^{\circ}\prime )\right]})}$$

where

(8) $$E_{g}=E_{G}-\left(\frac{V}{2}\right)$$

(9) $$E_{c}=E_{G}+\left(\frac{V}{2}\right)$$

where *V* is the generator-collector bias, the difference in applied potential between the collector and generator IDA electrodes for small values of *V* (typically <10 mV) for which *i*_gc_ changes linearly with *V* (33). Equation 7, which describes electrochemical gating measurements (28), indicates that *i*_gc_ and thus σ is dependent upon the gate potential, *E*_G_, the average of *E*_g_ and *E*_c_, which determines the oxidation state of the redox sites across the biofilm between the generator and collector electrodes. When *E*_G_ is equal to E°′, there is a 50/50 mix of reduced and oxidized redox sites, which enables the highest rate of long-distance electron transfer through the biofilm, and thus the largest *i*_gc_ (for a given small *V*), and thus maximum σ. Under this condition, equation 7 simplifies to:

(10) $$\sigma_{m}=\frac{n^{2}F^{2}DC_{T}}{4RT}$$

Combining generator-collector measurements with square-wave voltammetry as described above enables separation of effects of changes in the effective concentration of redox sites, *C*_T_, from changes in how effectively these redox sites are used in electron transfer through the biofilm, *D*, on the maximum biofilm conductivity, σ_m_, which occurs when *E*_G_ is equal to *E*°′. In this way, *C*_T_, *D*, and σ_m_ values were calculated from 3 biological replicates for each strain.
